# Supplementary figures and images for: 16S rRNA amplicon sequencing and antimicrobial resistance profile of intensive care units environment in 41 Brazilian hospitals
Source: Front Public Health. 2024 Jul 15;12:1378413. doi: 10.3389/fpubh.2024.1378413 (PMC11284946; doi:10.3389/fpubh.2024.1378413)

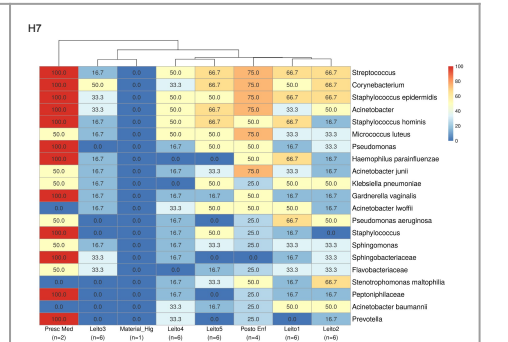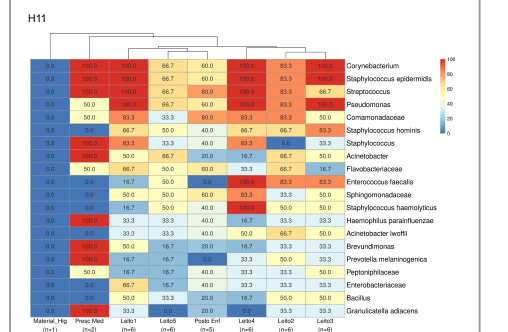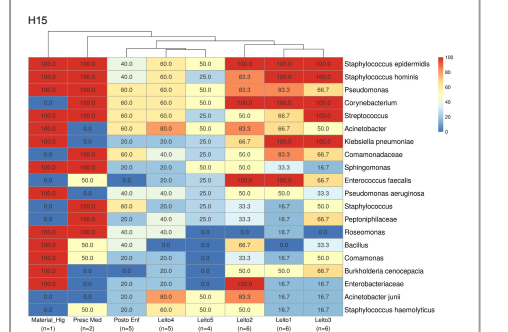

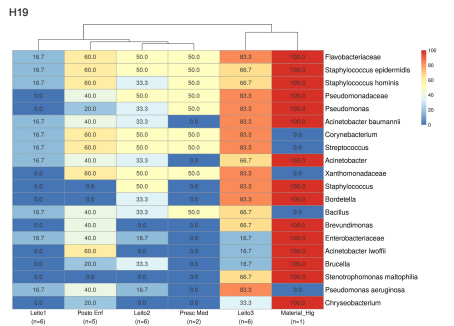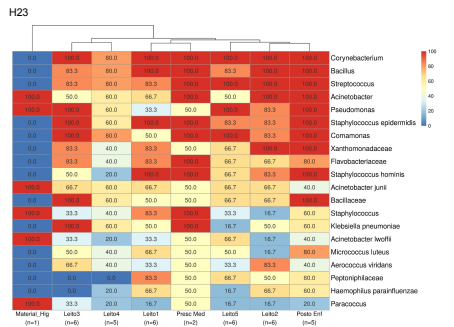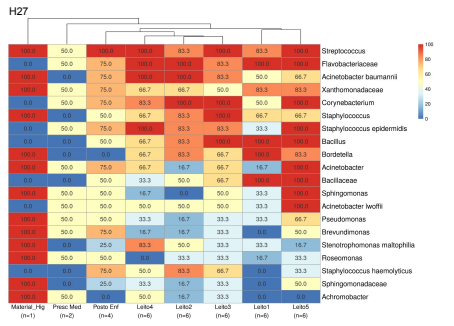

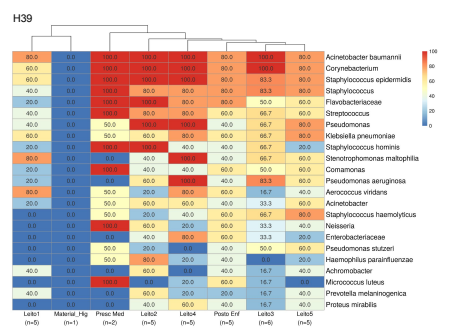

H41

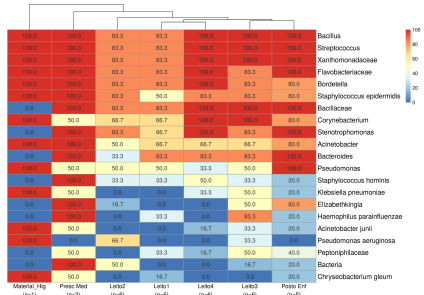

H2

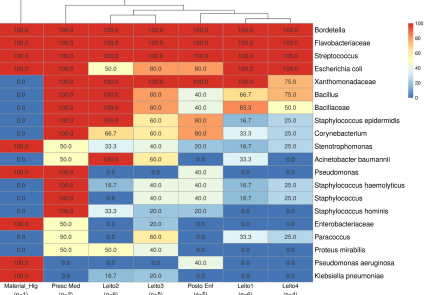

H43

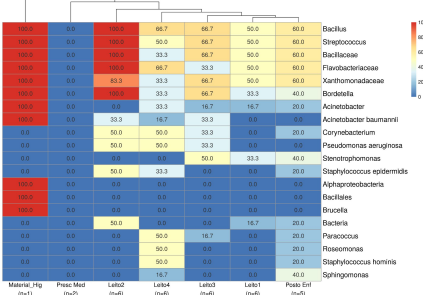

H45

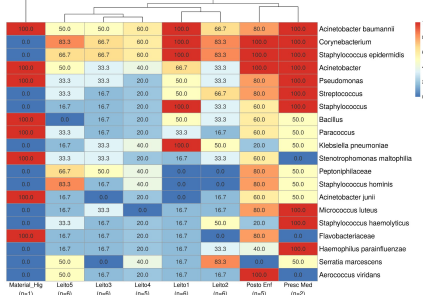

H47

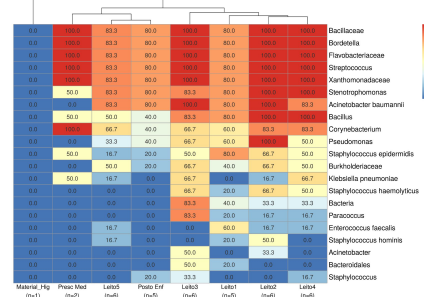

Supplement: Supplementary file 1 [file Data_Sheet_1.PDF]

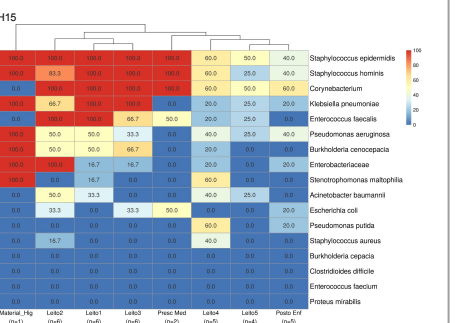

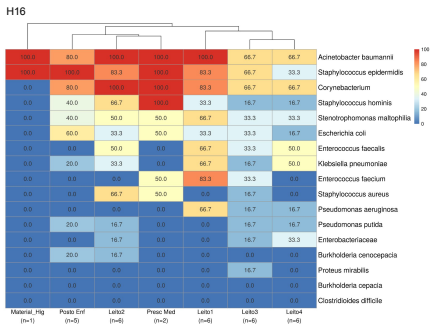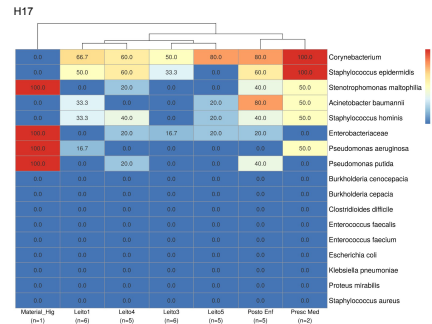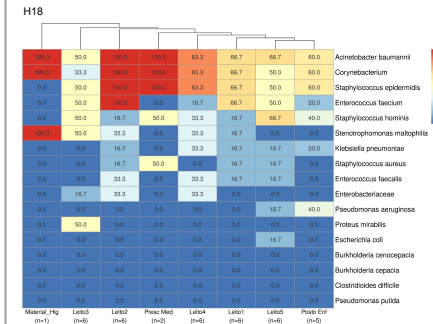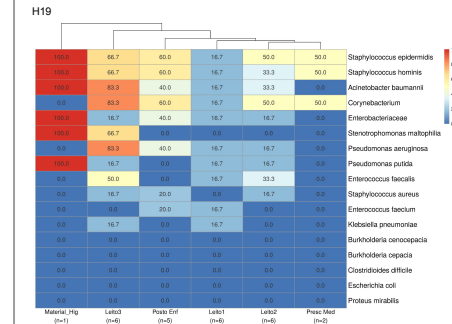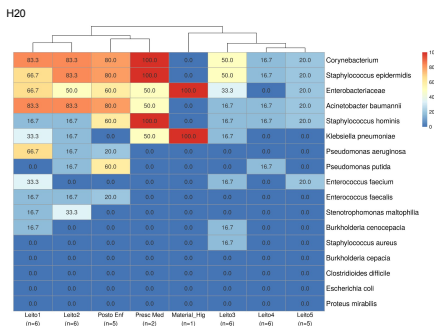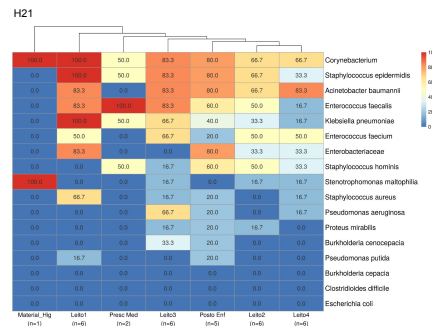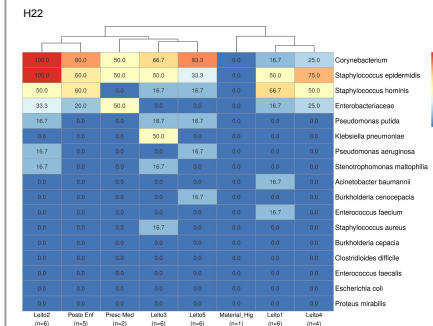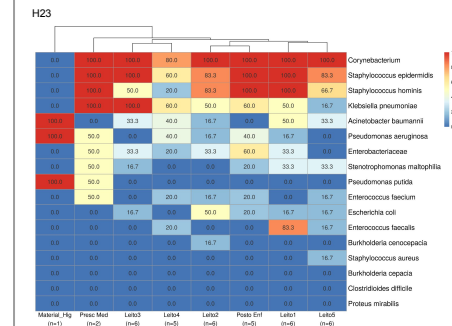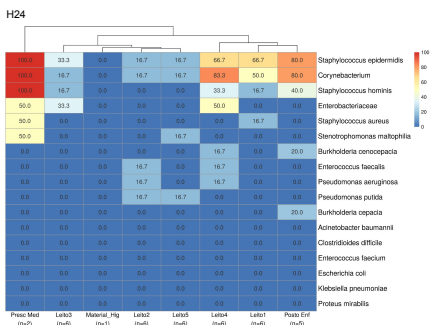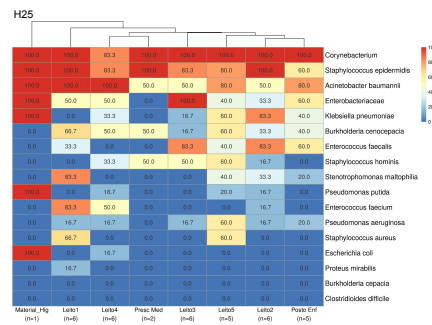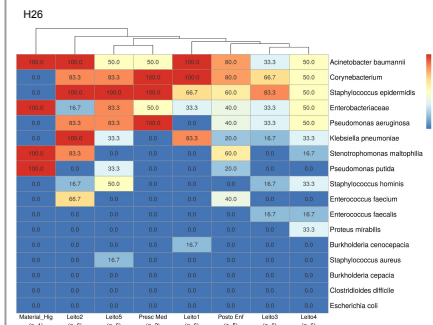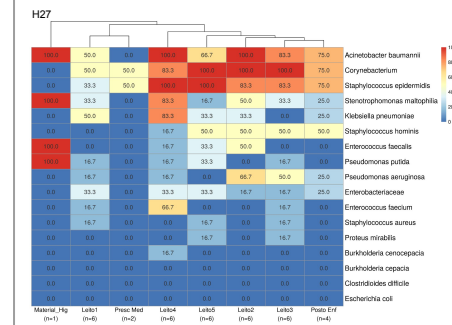



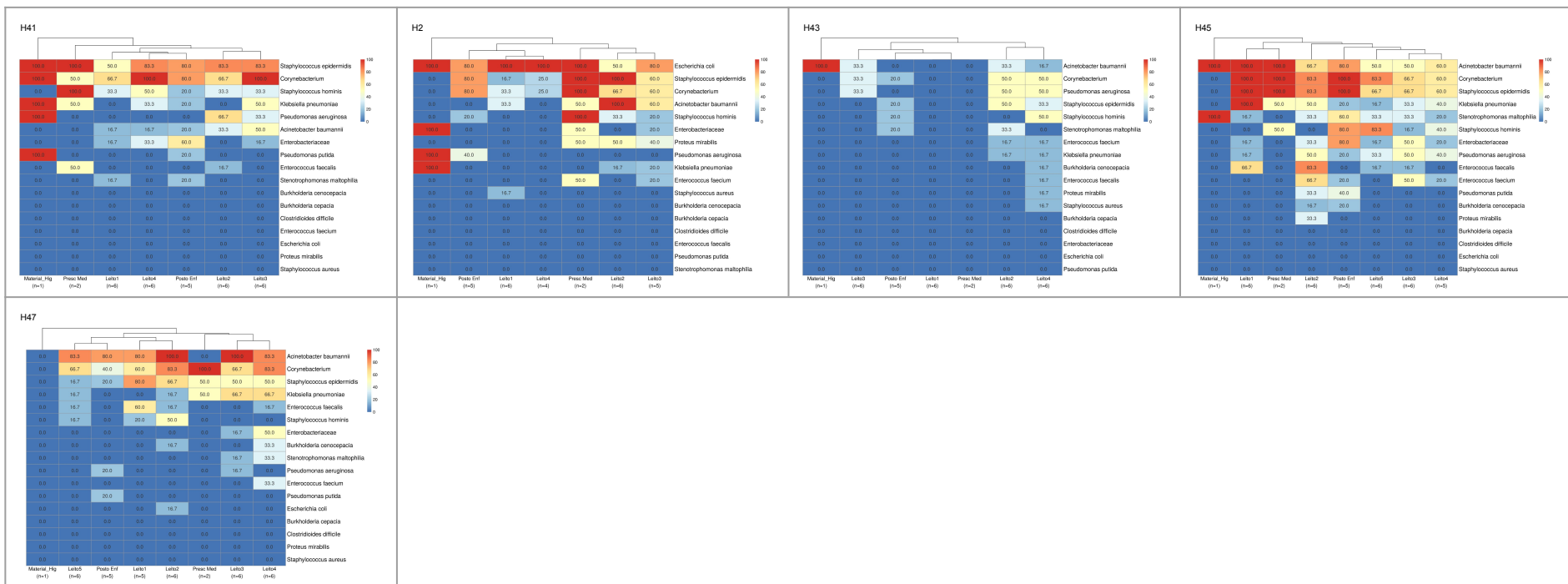

Supplement: Supplementary file 2 [file Data_Sheet_2.PDF]

A

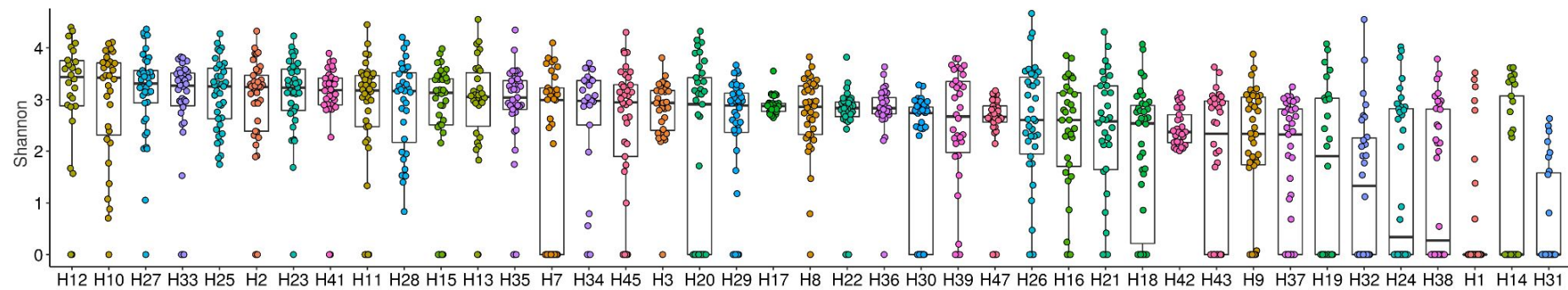

B

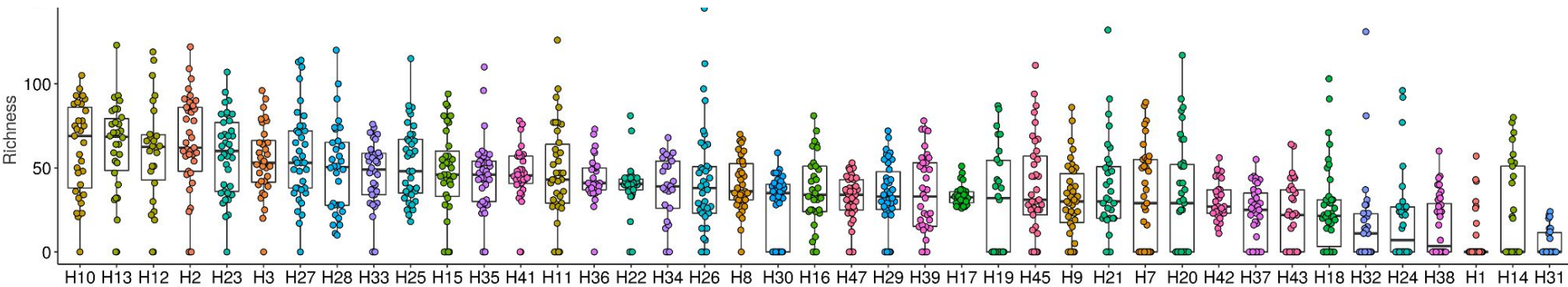

Supplement: Supplementary file 3 [file Data_Sheet_3.PDF]

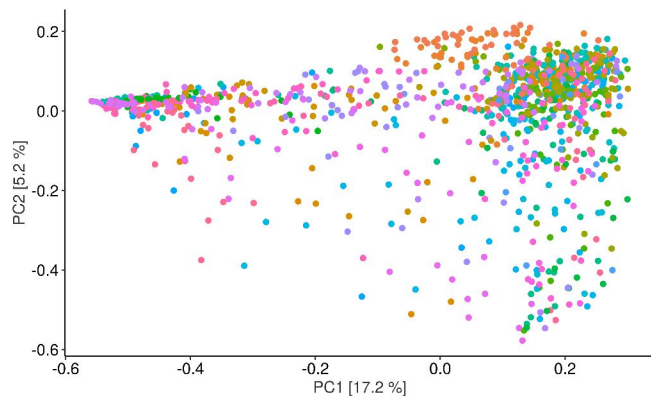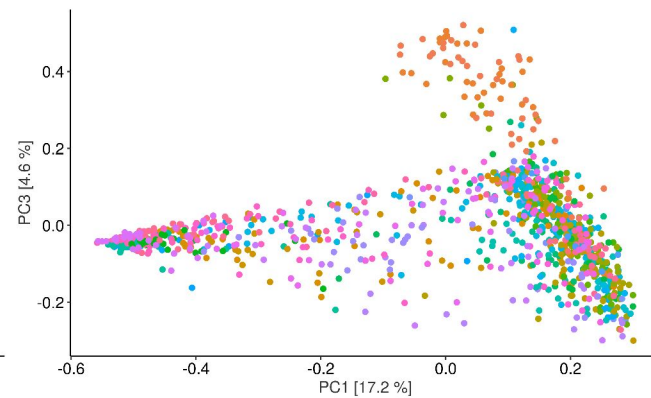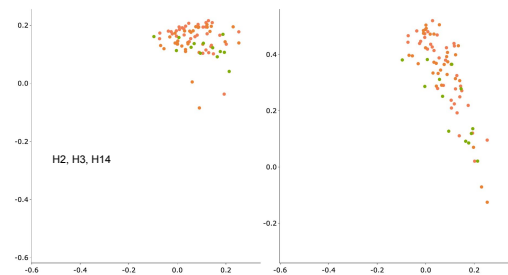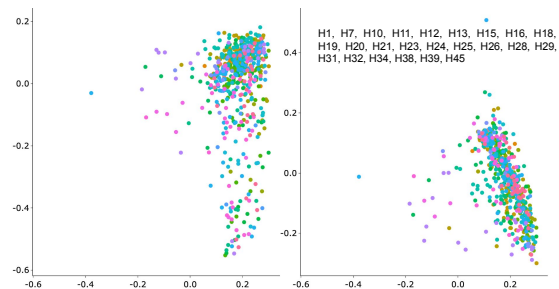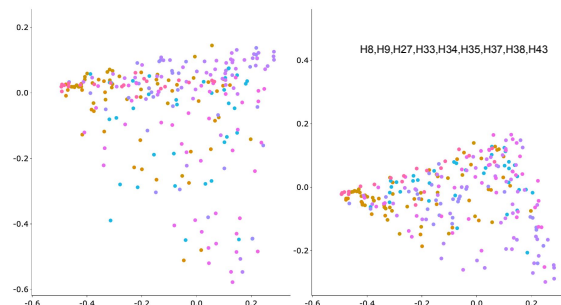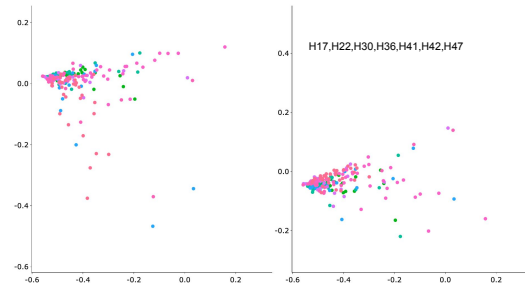

Supplement: Supplementary file 4 [file Data_Sheet_4.PDF]

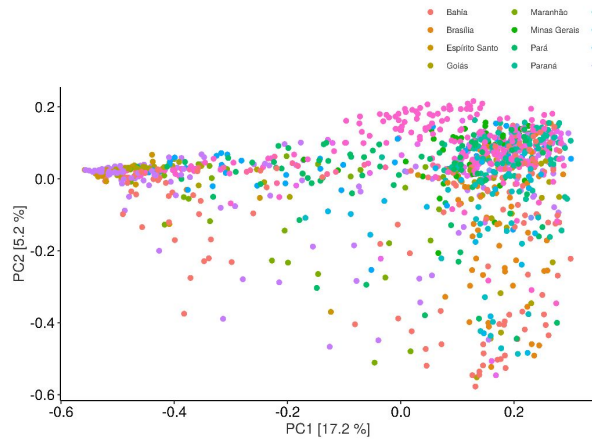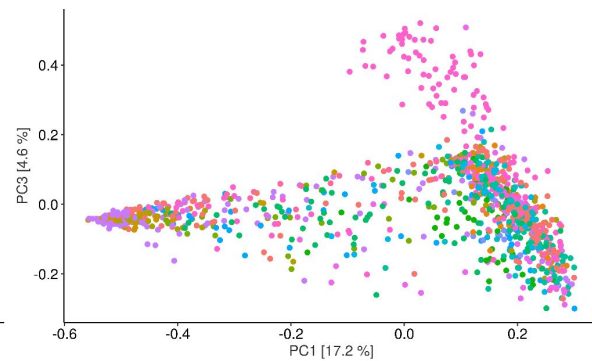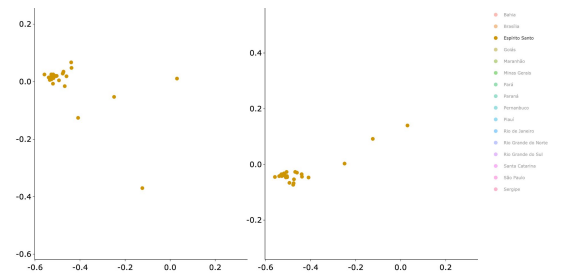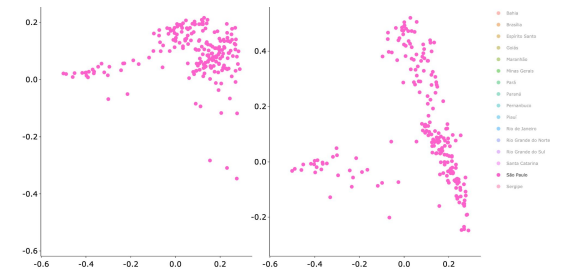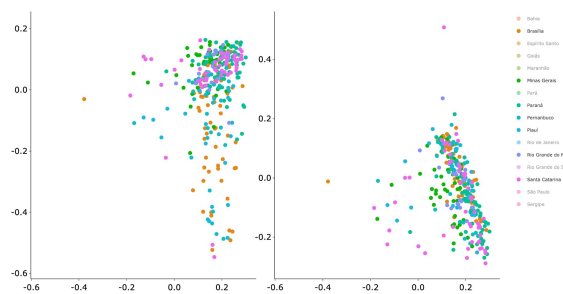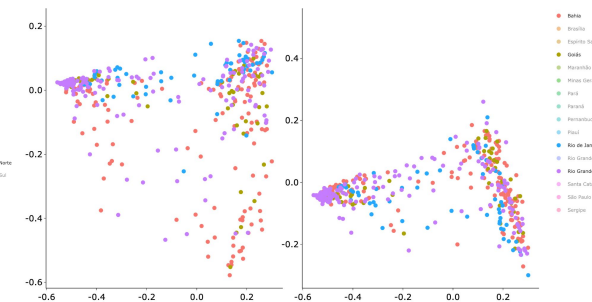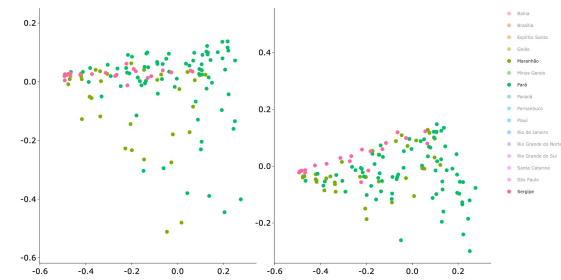

Supplement: Supplementary file 5 [file Data_Sheet_5.PDF]

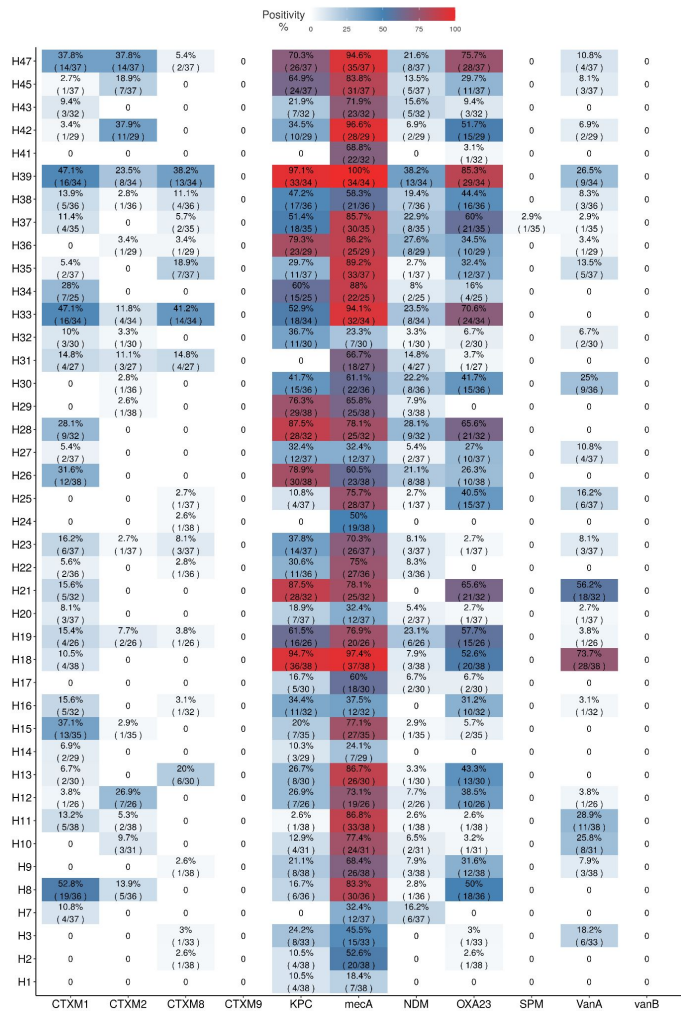

Supplement: Supplementary file 6 [file Data_Sheet_6.PDF]
